# Knowledge, attitude, practice and perceived barriers of nurses working in intensive care unit on pain management of critically ill patients: a cross-sectional study

**DOI:** 10.1186/s12912-022-00990-3

**Published:** 2022-07-26

**Authors:** Essa M. Sweity, Ahmad M. Salahat, Abd alrhman Sada, Ahmad Aswad, Loai M. Zabin, Sa’ed H. Zyoud

**Affiliations:** 1 Department of Cardiology, An-Najah National University Hospital, Nablus, 44839 Palestine; 2 College of Nursing and Midwifery, Women’s Federation Society, Nablus, 44839 Palestine; 3 Department of Nursing, An-Najah National University Hospital, Nablus, 44839 Palestine; 4 Department of Clinical and Community Pharmacy, College of Medicine and Health Sciences, An-Najah National University, Nablus, 44839 Palestine; 5 Clinical Research Center, An-Najah National University Hospital, Nablus, 44839 Palestine

**Keywords:** Pain management, Intensive care unit, Nurses, Knowledge, Practices, Perceived barriers, Critical Care Nursing, Palestine

## Abstract

**Background:**

Pain is a major obstacle and one of the main reasons people seek medical attention and is a frequent stressor for many clients in the intensive care unit (ICU). However, clients should not be left complaining, especially when solutions are available; each patient has the right to assess and manage their pain in the best way possible. Therefore, the objective of this study was to analyze nurses' knowledge, attitudes, and practice (KAPs) regarding pain management in Palestinian ICU settings and to determine the possible obstacles that may hinder effective and competent pain management for critically ill clients.

**Methods:**

This cross-sectional research was conducted online through social media. An approved questionnaire was used to assess KAPs and obstacles in pain treatment approaches for critically ill patients. Bloom’s cutoff points for adequate practice, appropriate knowledge, and a positive attitude were applied. IBM SPSS Statistics for Windows, Version 21.0 was used for analyses.

**Results:**

One hundred ninety-one nurses were approached, the majority of the participants in this investigation were males (*n* = 127, 66.5%), and the mean age of the study participant was 29 ± 7 (year). The overall knowledge score was 15, measured for median knowledge = 7 with an interquartile range (IQR) of 4–8, and higher scores indicate more knowledge about the management and control of pain. The total attitude score = 11, the median = 6, with an IQR of 5–7. The reluctance to prescribe opioids was 79.6%, the lack of proficiency in pain management knowledge was 78.5%, and rigorous controls over opioid use were 77.5%, which was the lion's share of commonly recognized hurdles. The overall practice score was 10, with a median of 5.0 with an IQR of 3.0 to 6.0, and nurses revealed that they would evaluate all the steps involved in pain management in each round they have.

**Conclusions:**

This research reveals a knowledge, attitude, and practice gap among the working nurses. Therefore, adequate and efficient plans must be aimed at ICU nurses to foster the level of knowledge and direct attitudes toward pain control through applicable interventional programs.

## Background

In the care of critically ill clients, pain is a serious obstacle. Pain is defined as an unpleasant sensory and emotional experience associated with, or similar to, actual or potential tissue damage [1]. Additionally, pain is a major obstacle and one of the main reasons people seek medical attention. It is a frequent stressful manifestation for numerous clients in the intensive care unit (ICU). However, many cases related to mechanical ventilation and the management of those patients were considered a vital aspect of the clinical scope. Furthermore, 45 to 82% of patients in the ICU experienced moderate to severe pain during rest and procedural interventions [2]. Ethically, clients should not complain, especially when available solutions [3]. Inaccurate evaluation of pain and maintaining clients suffering from pain can harm quality of life, leading to high morbidity and mortality. Therefore, managing pain is considered a crucial part of controlling the quality of life. As several studies revealed, nurses’ knowledge and attitudes contribute to the pain management journey and remarkable decision-making skills on the clinical site [4, 5]. Previous research has also found that nurses lack pharmacological awareness, particularly regarding the use and/or abuse [6–9]. The lack of courses in nursing schools to improve pain management can be attributed to the knowledge gap among nurses.

However, earlier literature indicated the sufficiency of knowledge and attitudes in managing pain effectively and the lack of nurses’ ability to perform pain assessment and management [10]. Pain management skills among nursing workers are considered vital for nursing care, where healthcare workers can deal with pain in the most effective way [11]. In general, nurses earn pain management beliefs from thorough knowledge, where negative outcomes are possible, especially when knowledge is distorted, insufficient, or wrong. Many pain concerns, such as incorrectly judging suffering to be less intense than it is, a lack of understanding of painkiller medications, and the assumption that clients misrepresent their discomfort [12], can contribute to inappropriate pain treatment.

In ICUs, pain assessment documentation is suboptimal, particularly for patients who cannot self-report or receive higher opioid doses. In addition, tools to improve pain assessment and documentation are still needed. The shorter hospital stays, greater client satisfaction, and reduced reported mortality and morbidity are positive outcomes of enhancing nursing knowledge, approach, and attitude toward pain treatment [13, 14]. Pain histories, therapies, and re-assessment measures of the client must be documented for better research and practice. However, there has not been much study documenting pain evaluation and management in intensive care. Many retrospective studies revealed that pain documentation is generally imprecise and undertreated pain [15, 16]. The quality of nursing documentation on pain evaluation and management is still insufficient worldwide. This inadequacy can be exacerbated by a failure to use pain flow sheets in clients' health records [16].

Nursing knowledge and attitudes toward pain assessment and management are not well established in Palestine, and more studies are required on this critical topic. Therefore, this study aims to examine nurses' knowledge, attitudes, and practice regarding pain in intensive care settings in the state of Palestine and determine obstacles to the successful management success among critical care clients.

## Methods

### Study design

A cross-sectional, observational, and descriptive study was conducted among Palestinian nurses.

### Population and study setting

This research was carried out in the West Bank of Palestine of targeted nurses with various educational levels serving in a critical care setting, such as master’s degree nurses, registered nurses (RN), and licensed practical nurses (LPN).

### Study sample and sampling technique

An online questionnaire was used to collect data for this study. The questionnaire items were required to complete, not optional, data collected from 01 June to 01 October 2021. This was the only possible option to distribute the surveys during government lockdown and transportation prohibitions. Google Forms (https://www.google.com/forms) were used to generate the questionnaire. The sample size needed for the present study was calculated using a commonly used calculator to calculate sample sizes. The calculator can be accessed online through the link (www.raosoft.com). The calculator estimates sample sizes using Daniel’s formula. The number of nurses was estimated at a 95% confidence interval (CI) and tolerating a default margin of error of 5%. For this study, 191 respondents were determined.

### Approval of ethics

The Institutional Review Board (IRB) of An-Najah National University (NNU) in Palestine allowed this current research.

### Tools of study

The questionnaire was adapted using standard methodology, including literature information review, pilot studies, group discussion, evaluation by domain experts, and validation of the items included in the questionnaire used [9, 17–19].

Face and content validity of the final questionnaire was discussed and judged by four expert’s academicians in the related field for assessing the completeness, appropriateness, meaning of terms, logical sequence of the statements, and organization and the accuracy. Some questions were modified as recommended. The final questionnaire was initially piloted among fifteen nurses before the start of the study to evaluate clarity, and time considerations; only minor modifications were needed. The final English document was translated into Arabic to ensure that nurses and staff understood the questionnaires fully. Internal consistency reliability for knowledge scale was good, with Cronbach’s α = 0.70.

The last approved questionnaire form consisted of five sections that assessed nurses' demographic and sociological variables and their attitude, practice, and perceived barriers. A total of 44 closed- and open-ended questions have been introduced. Six questions about ages, sex, workplace, work area, working experiences, educational certification, and whether previous pain management courses had been attended were asked in the first sociodemographic part.

The second section evaluated nurses' knowledge of pain for critical care patients using 15 questions. True and false questions were used to examine respondents' knowledge of the principles of pain and their ability to identify it from other sources of pain and patient evaluation in the ICU. Subsequently, for the 15 knowledge questions, a mark was assigned to every correctly given response, resulting in a knowledge score, and the increased scores suggest that nurses have greater knowledge [17].

The third section evaluated nurses' attitudes towards pain management of critically ill patients via eleven questions and answered choices: "yes", or "no". Each successful answer to the question in the 11 knowledge questions receives one point. The attitude score was calculated by summing up the points assigned to each successfully answered question on the 11 attitude questions. A higher score indicates a positive attitude [18].

The fourth section addressed perceived challenges in caring for critically ill individuals. Clients, healthcare workers, and difficulties related to the healthcare system were divided into three groups in the questions on perceived obstacles.

The last section of the survey inquired about pain assessment documentation practices in critical care clients, the types of pain assessed pain, and whether nurses recorded pain in the patient’s medical file after each evaluation procedure. ‘Agreement statements’ inquiries have been used in the last two sections [9].

The cutoff point for this study was by categorizing nurses into two groups using median utility indexes; whoever obtained a score higher than the median was labeled as having sufficient knowledge, good attitude, and practices. In contrast, anyone who obtains a score lower than the median was labeled with insufficient knowledge, with poor attitude and practices [20].

### Statistical analysis

Characteristics and questions about their knowledge, practices, and perceptions of obstacles were described using descriptive analysis. Data normality was tested using the Kolmogorov–Smirnov test. The data was not normally distributed. Thus, nonparametric statistical tests were used. Scale data are expressed as the median (quartile 1 [Q1]—quartile 3 [Q3]). The relation between knowledge score and participant characteristics was revealed. IBM SPSS version 21.0 was used for statistical analysis. Percentages and frequencies, median with interquartile range, are some of the statistics used to represent the data. At *p* < 0.05, the Kruskal–Wallis and Mann–Whitney U tests were used. The internal consistency and reliability of the knowledge scale were assessed using Cronbach's alpha coefficient. The internal consistency coefficients indicated acceptable reliability of the tool (Cronbach's α = 0.70).

## Results

### Socio-demographic features

Our study was carried out on 191 patients. As shown in Table 1, 66.5% (*n* = 127) of the participants were male. Nurses with less than two years of experience amounted to 47 (24.6%). The majority (*n* = 102; 53.4%) of the participants practiced in private hospitals. Of the 191 participants, the vast majority (*n* = 122; 63.9%) of the participants were registered nurses. The lowest proportion of nurses working area in the MICU (*n* = 25; 13.1%). The majority (*n* = 129; 67.5%) of the participants did not enroll in a previous course in pain management. Table 1 shows the social demographics of the nursing participants in this study.

**Table 1:** Demographics and characteristics associated with nurses' knowledge, attitude, and practices towards pain management of critically ill patients

Demographics	Number (%)	Median knowledge [Q1- Q3]	*P* value	Median Attitude [Q1- Q3]	*P* value	Median Practices [Q1- Q3]	*P* value
**Sex**							
Male	127 (66.5)	6 [4-8]	0.160^a^	6 [5-7]	0.168^a^	5 [3-6]	0.168^a^
Female	64 (33.5)	7 [5-8]		6 [5-7]		6 [3-7]	
**Age (year)**							
> 25	38 (19.9)	7 [5-8]	0.412^b^	6 [5-7]	0.476^b^	3 [2-6]	< 0.001*^b^
25–29	89 (46.6)	7 [4-8]		6 [5-7]		4 [3-7]	
30–34	25 (13.1)	6 [3–7.5]		5 [5-6]		6 [5,6]	
> 35	39 (20.4)	6 [4-8]		6 [5-7]		6 [5-7]	
**Experience (year)**							
< 2	47 (24.6)	7 [5-8]	0.713^b^	7 [5-7]	0.816^b^	4 [2-6]	< 0.001*^b^
3–5	62 (32.5)	7 [4-8]		7 [5-7]		4 [3-7]	
6–9	38 (19.9)	6 [4-8]		6 [5-7]		6 [3-7]	
< 10	44 (23)	6 [3-8]		6 [5-7]		6 [5-7]	
**Hospital type**							
Government Hospital	89(46.6)	7 [2.5–8.5]	0.667^a^	6 [5-7]	0.765^a^	5 [5-6]	0.361^a^
Private Hospital	102 (53.4)	7 [5-8]		6 [5-7]		5 [5-6]	
**Work area**							
PICU	31 (16.2)	8 [4-8]	0.183^b^	6 [5-7]	0.193^b^	5 [3-7]	0.688^b^
MICU	25 (13.1)	7 [4-8]		6 [5 – 7.5]		6 [2 – 6.5]	
CICU	65 (34.0)	6 [4-8]		6 [5-7]		6 [5-6]	
SICU	27 (14.1)	7 [5-8]		6 [5-7]		5 [3-6]	
General ICU	43 (22.5)	5 [2-8]		5 [5-7]		4 [3-6]	
**Level of education**							
Master in Nursing	41 (21.5)	8 [6-10]		7 [6-8]		6 [4-7]	
Registered nurse	122 (63.9)	7 [5-8]	< 0.001*^b^	6 [5-7]	< 0.001*^b^	5 [3-6]	0.009*^b^
Practical nurse	28 (14.7)	5 [3-7]		5 [5-6]		5 [3-6]	
**Did you take a previous course on pain management?**							
Yes	62 (32.5)	7 [5 – 8.5]	0.002*^a^	6 [5-7]	0.003*^a^	7 [6-7]	< 0.001*^a^
No	129 (67.5)	6 [3-8]		5 [5-6]		4 [3-5]	

*Abbreviations*: *PICU* Pediatric ICU, *MICU* Medical ICU, *CICU* Cardiac ICU, *SICU* Surgical ICU

^a^*p*-value is calculated using the Mann–Whitney U test

^b^*p*-value is calculated using the Kruskal–Wallis test

### Knowledge about pain management in critically ill patients

The knowledge regarding pain management has been evaluated using 15 questions, each with a numerical weight assigned, where “Yes” = 1 “correct answer”, “No” = 2 “incorrect answer” and “I don't know” = 3, as shown in Table 2.

**Table 2:** Nursing knowledge about pain management of critically ill patients

**Asked questions**^b^	**True n (%)**^a^	**False n (%)**^a^	**I don’t know n (%)**
1. Vital signs are always reliable indicators of the intensity of pain in a patient	121(63.4)	**59(30.9)**	11(5.8)
2. Important to assess pain among patients with Glasgow coma Scale > 8	**145(75.9)**	31(16.2)	15(7.9)
3. Patients who can be distracted from pain usually do not have severe pain	118(61.8)	**56(29.3)**	17(8.9)
4. Aspirin and other non-steroidal anti-inflammatory agents are ineffective in treating pain in the ICU	93(48.7)	**69(36.1)**	29(15.2)
5. Respiratory depression rarely occurs in patients who have received stable doses of opioids over months	**97 (50.8)**	50 (26.2)	44 (23)
6. Combining analgesics that work by different mechanisms (for example, combining an opioid with an NSAID) may result in better pain control with fewer side effects than using a single analgesic agent	**115 (60.2)**	33 (17.3)	43 (22.5)
7. The usual duration of analgesia of 1 to 2 mg of morphine IV is 4 to 5 h	104 (54.3)	**33 (17.3)**	54 (28.3)
8. Opioids should not be used in patients with a history of substance abuse	89 (46.6)	**44 (23)**	58 (30.4)
9. Morphine has a dose ceiling (i.e., a dose above which 2 greater pain relief can be obtained)	92 (48.2)	**37 (19.4)**	62 (32.5)
10. Pethidine can be prescribed for chronic pain safely	76 (39.8)	**59 (30.9)**	56 (29.3)
11. Important for assessing pain for patients at end-of-life	**116 (60.7)**	24(12.6)	51 (26.7)
12. If the patient's source of pain is unknown, opioids should not be used during the pain evaluation period, because this could mask the ability to correctly diagnose the cause of pain	103 (53.9)	**32 (16.8)**	56 (29.3)
13. Benzodiazepines are not effective pain relievers unless the pain is due to muscle spasms	**75 (39.3)**	46 (24.1)	70 (36.6)
14. The recommended route of administration of opioid analgesics for patients with persistent cancer-related pain is: (oral)	**83 (43.5)**	41 (21.5)	67 (35.1)
15. The recommended route of administration of opioid analgesics for patients with a brief severe sudden onset, such as trauma or postoperative pain, is: (intravenous)	**100 (52.4)**	33 (17.3)	58 (30.4)

^a^The correct answers are highlighted in bold

^b^These questions were adapted from previous studies Toba et al. [9] Al-Sayaghi [19] Ufashingabire [17]

The median was 7.0 with an interquartile range of 4.0 to 8.0. 3.4% of the participants acknowledged that vital signs are always trustworthy predictors of the level of pain of a client.

The result showed insufficient (poor) knowledge of pain in the ICUs, having a median knowledge score of 7 out of 15. When respondents were asked ‘if pain is important to evaluate in patients with a Glasgow coma scale of more than 8’, 75.9% of respondents correctly agreed. Of the group, 61.8% incorrectly agreed that ‘Clients who might be distracted from their pain are less likely to be in intense pain’. Furthermore, knowledge of the ‘analgesic effect with 1–2 mg of intravenous morphine IV that generally last 4–5 h” showed a response variation as 54.3% agreed incorrectly, the same variation when the respondent asked about ‘morphine has a dose ceiling (a level above which more pain relief is provided).’ 48.2% agreed incorrectly.

Only 23% of the respondents correctly agreed with the statement ‘opioids should not be administered to people with a history of drug misuse’, and 30.9% correctly answered ‘for chronic pain, pethidine can be prescribed safely’. Knowing that 60.7% of nurses agreed that “It is crucial to perform a pain assessment in people who are near the end of their lives” and only 16.8% correctly reported that “Opioids should not be taken at the time of pain evaluation if the causes of the client's pain are unclear, as this could obscure the capability to appropriately evaluate the reason for the pain”.

Regarding the point “If muscle spasms cause pain, benzodiazepines are ineffective pain medications”, 39.3% of the nurses participated agreed and 43.5% of the nurses agreed correctly with the point of “Oral rout is the appropriate method to administer opioid painkillers to clients with chronic cancer-related pain ", and “the administration of opioid drugs intravenously is the preferred method for people with short, intense, and abrupt onset pain, for example postoperative and traumatic pain”. More than half of the enrolled participants, 52.4%, correctly.

Table 1 investigates the relationship between sociodemographic factors and the Mann–Whitney U and Kruskal–Wallis. tests were used. Consequently, the median knowledge score) among women and men nurses was not significant statistically. Furthermore, no relationship was found between age, experience, location of employment, and work area in the level of knowledge.

Although there was a statistically significant with the highest level of education, as the level increased, the level of pain in the ICU improved. Furthermore, the participant who had previously taken a course on pain management showed better pain management for ICU patents.

### Attitude towards pain management of critically ill patients

Nursing attitudes toward pain management for critically ill patients were evaluated using 11 questions and numerical values were assigned to each item, one for ‘yes’ and 2 for ‘no’ (Table 3).

**Table 3:** Nurses’ attitude towards pain management of critically ill patients

**Asked questions**^b^	**Yes n (%)**^a^	**No n (%)** ^a^
1. Do you think that patients who can be distracted by pain usually do not have severe pain	108(56.5)	**83(43.5)**
2. Do you think that the patient may sleep despite pain	**94(49.2)**	97(50.8)
3. Do you think elderly patients cannot tolerate opioids for pain relief?	83(43.5)	**107(56)**
4. Do you think patients should be encouraged to endure as much pain as possible before using an opioid?	90(47.1)	**101(52.9)**
5. Do you think that children less than 11 years old cannot reliably report pain, so clinicians should rely solely on parents for the child's pain intensity?	78 (40.8)	**113 (59.2)**
6. Do you believe that giving narcotics on a regular schedule is preferred over the PRN schedule for continued pain?	**89 (46.6)**	102 (53.4)
7. Do you think that giving patients sterile water by injection (placebo) is a useful test to determine if the pain is real?	105 (55)	**86 (45)**
8. Do you think that lack of pain expression does not mean lack of pain	**87 (45.5)**	104 (54.5)
9. Do you think that analgesics for postoperative pain should initially be administered around the clock on a fixed schedule	**124 (64.9)**	67 (35.1)
10. Do you believe that patients' spiritual beliefs can lead them to think pain and suffering are necessary?	**117 (61.3)**	74 (38.7)
11. Do you think that the most accurate judge of the intensity of the patient’s pain is the patient	**133 (69.6)**	58 (30.4)

^a^The correct answers are highlighted in bold

^b^These questions were adapted from previous studies Toba et al. [9] Al-Sayaghi [19] Ufashingabire [17]

The median was 6.0 with an interquartile range of 5.0 to 7.0. The result showed poor attitude toward pain management, having a median knowledge score of 6 out of 10. Table 4 shows the distribution of the responses for all attitudes-related questions, showing that 43.5% did not think correctly that “Clients who can be diverted from their pain are typically not in severe pain, and about half of the participants correctly think that ‘Regardless of their pain, patients can sleep’.

**Table 4:** Perceived barriers to pain management in critically ill patients

Asked questions		Number of nurses (%)
Barriers related to medical staff	1- Inadequate pain assessment	111(58.1)
	2. Inadequate experience with pain control	119(62.3)
	3. Insufficient knowledge about pain control	133(69.6)
	4. Time constrains	133(69.6)
	5. Reluctance to prescribe opioids	152 (79.6)
	6. Insufficient communication with patient	149 (78)
Barriers related to patient	7. Reluctance to report pain	146(76.4)
	8. Patient-related: Insufficient communication with medical personnel	146 (76.4)
	9. Patient-related: Financial constraints	134 (70.2)
	10. Patient-related: Insufficient knowledge of pain control	150 (78.5)
Barriers related to the health care system	11. Related to the health care system: strict regulation of opioids	148 (77.5)
	12. Related to the health care system: inadequate staffing	137 (71.7)
	13. Related to the health care system: Limited stock of different types of opioids	141 (73.8)
	14. Related to the health care system: ICU pain management is not considered important	112 (58.6)
	15. Related to the health care system: Medication and intervention costs	135 (70.7)

A significant number (56%) of the participating nurses stated that ‘for pain treatment, opioids are not well tolerated by the elderly’ Concert with 59.2% agreed that ‘Because children under the age of 11 cannot consistently express pain, physicians should depend only on parents to determine the severity of child's suffering’ while 46.6% believed that ‘for persistent pain, it is preferable to provide narcotic drugs on a scheduled basis rather than on PRN bases. On the contrary, while asked about ‘Managing the client’s sterile saline solution (placebo) is a good way to see whether the discomfort is genuine or not’, only 45% correctly answered.

When asked ‘The absence of pain expression does not imply the absence of pain’, up to 45.5% (*n* = 87) were correctly answered, the majority of the participants correctly acknowledged that ‘postoperative analgesia must be administered regularly, round the clock’, according to 61.3% of the respondents who thought that the spiritual beliefs could make them to believe that suffering and pain are essential’, also the vast majority state that ‘The client himself/herself is the best judge of how severe the pain is’.

Table 1 shows the apparent and significant relationship among the attitudes of nursing staff and two demographic factors, the level of education, and the previous course of pain (Kruskal–Wallis and Mann–Whitney U tests). As the level of education increased, the attitude improved. Additionally, the participant who took a previous course in ICU pain management showed better pain management. On the contrary, there was no relationship between age, sex, experience, place of work, area of work, and attitude level.

### Perceived barriers to pain management

The most common obstacle related to medical personnel was the reluctance to prescribe opioids (79.6%). In terms of client aspects, the most prominent impediment was a lack of understanding of pain (78.5%). Finally, according to the respondents, the most prevalent constraint related to the health care system is strict control of opioid use (77.5%), as shown in Table 4.

### Practices in the management of critically ill patients

Pain assessment practices and documentation on pain management of critically ill patients were evaluated using six questions containing five criteria and numerical values for each response (ie 1 for yes and 2 for no) (Table 5).

**Table 5:** Pain assessment practices and documentation for pain management of critically ill patients

Type of practice	Number of nurses (%)
1. Occasion of pain assessment	
In every round	75 (39.3)
On selected occasions	95 (49.7)
On rare occasions	21 (11)
2. Which of the following items do you check when assessing pain?	
Location	139 (72.8)
Quality	139 (72.8)
Related factor	132 (69.1)
Severity	164 (85.9)
Timing	118 (61.8)
3. Documentation of pain assessment	152 (79.6)

The median was 5.0 with an interquartile range of 3.0–6.0. The result showed poor practices toward pain management, having a median knowledge score of 5 out of 9. Around half of the nurses who participated in the research would occasionally assess pain. (49.7%), but 39.3% of the participants reported feeling pain every round. Nurses also said that they examine all pain characteristics throughout the evaluation procedure, with severity being the most commonly rated aspect. Furthermore, most nurses stated that they maintain a record of assessed pain.

The results shown in Table 1 did not reveal any significant relationship between sociodemographic characteristics (sex, place of work, area) and pain assessment practices and documentation for the treatment of pain of critically ill patients (Kruskal–Wallis test and Mann Whitney U). However, there were statistically significant differences in age and experience; As practice improved and progress, age and experience increased. Although the result showed obvious significance in the level of education, practices improved as the level of education increased. Furthermore, another significant showed with previous courses in line with the good practice of nurses in pain management with a *p*-value (< 0.001).

### The correlations between knowledge, attitude, and practice scores on pain management of critically ill patients

The KAP scores for the questionnaire used were not normally distributed, as shown by the Kolmogorov–Smirnov test. Therefore, the correlation between elements was assessed, and a positive correlation was discovered between the KAP scores. Spearman rank correlation (r_s_) was used to calculate the correlation values among the KAP score calculations. A significant modest positive correlation was shown between the knowledge and attitude scores of the respondents about pain management of critically ill patients (*r* = 0.967, *p* < 0.001). Furthermore, a significant modest positive correlation was demonstrated between the knowledge and practice scores of the respondents in the management of critically ill patients (*r* = 0.144, *p* = 0.048). Finally, there was a significant modest positive correlation between attitude and practice scores in pain management of pain in critically ill patients (*r* = 0.148, *p* = 0.041). As shown in Table 6.

**Table 6:** Correlations among KAP

Variable	rs	*P*-value
The Knowledge and attitude	0.967	< 0.001*
The Knowledge and practice	0.144	< 0.048*
The Attitude and Practice	0.148	< 0.041*

^*^p < 0.05 considered to have Statistical significance

## Discussion

This study was designed to evaluate and assess the knowledge levels, attitudes, and perceived barriers of nursing staff working in ICUs to manage the pain of critically ill clients.

According to our findings, Palestinian nurses had a level of insufficient knowledge of pain in the ICUs, having a median knowledge score of 7 out of 15 and a knowledge deficit mostly in the duration of the opioid dose, the risk of addiction, respiratory depression, and the effects of the opioid limit.

The current results are consistent with previous studies, in which nursing staff had the fewest acceptable responses to inquiries concerning opioid administration and opioid addiction and the knowledge gap in pain physiology and pharmacology [21–23]. These results align with Palestinian research that found that nursing staff in critical care settings in Palestine had insufficient knowledge of pain control and reflect this. The degree of knowledge they have obtained may affect their pain management practices. Consequently, improving the knowledge and attitudes of nursing staff about pain treatment is critical to improving quality of life [24, 25].

In addition, another study consistent with the knowledge questions and how they are answered stated that nurses did not have adequate knowledge in the study by Yava et al. [26] and Wang and Tsa [27]. Another study by Kahsay and Pitkäjärvi agreed with our result on emergency nurses with a severe knowledge deficit [28]. Toba et al. also concluded that Palestinian nurses had a fair level of knowledge about pain in cancer patients; however, many cancer patients were involved in ICU care [9].

In a study measure, the knowledge of the Jordanian nurse has been shown to have inadequacies in managing and assessing of pain [29]. However, there is insufficient knowledge, but it is attributable to the academic preparation provided to nurses during their academic journey. This explains the level of education for nurses, since when the level of education increases, the knowledge of pain control increases, as shown in our result [29]. This observation is consistent with a study conducted in Turkey where nurses with a master's degree or higher educational level and those who took a postgraduate course on pain control have a statistically significant higher knowledge score than nurses with an associate degree [26]. Similarly, the findings are consistent with the research findings carried out at the Gondar Comprehensive Specialized Hospital [30]. Although the primary areas of knowledge deficit were similar to those found in the Korean study, the knowledge tended to be lower [31].

Training related to ICU patient pain management for the last two years strongly associated with having adequate knowledge about pain parallel to an institutional-based cross-sectional study conducted in Mekelle [32] and Amhara referral hospitals in northern Ethiopia [33]. This could be due to a lack of preparation for nursing programs and continued education. The lack of such programs can result in a lack of understanding of pain assessment and management. Educational programs for nurses to increase their knowledge and abilities in pain management have received much attention in the literature. According to Abdalrahim et al., the mean knowledge score increased after participating in a pain management program [34]. Similarly, after a six-hour training session on pain evaluation and management, Qadire reported a considerable increase in nursing knowledge and attitude [35], also Samarkandi (2018) emphasizes the need for more training and education on pain management in Middle East countries [18]. In line with our findings, Academic credentials with further courses related to pain management linked to knowledge, attitudes, and practices on pain management were significantly different among nurses [24]. On the other hand, Nimer & Ghrayeb (2017) showed that level of education does not make any difference to knowledge and attitudes in relation to pain management [25].

Our research revealed a poor attitude towards pain management. This finding was consistent with research carried out in Palestine showed a negative attitude. Although in near-culture, Issa et al., 2017 reported that almost half of critical care nurses had a poor attitude toward pain treatment while working with ICU patients [3]. Other studies evaluating Saudi nurses’ knowledge and attitudes toward pain management revealed that inadequate knowledge and negative attitudes were found and nurses continue to under-assess and undertreat pain [3, 19]. Although studies carried out in Zimbabwe [21], Western Canada [36], and Jimma, western Ethiopia [37] are surprising that different cultures have similar attitudes.

This result was logical with a different population lower than those of the research conducted in Malaysia [38] and Uganda [33], during which the survey respondents had a good attitude towards pain control. On the other hand, our findings were higher than those of the investigations carried out in the United States [39] and Hong Kong, China [40]. This variation was attributed to cultural factors, which reflected a possible variation in the level of coverage of pain management topics in undergraduate education in different countries.

Impressively, during the current investigation, more than half of the participants reported that injecting clients with sterile saline/water (that is, placebo) is an effective test to determine whether or not their pain is genuine. It is unethical practice for patients suffering from pain. However, when asking about ‘Patients should be prompted to tolerate the most discomfort as possible before resorting to pain killers’, around half of the participants reported ‘No’ as it is unethical to give a placebo. However, ICU nurses must understand that it is not advantageous for a client in pain to examine the cause during the pain evaluation procedure [3].

Furthermore, study participants identified significant obstacles that limit appropriate pain control in critically ill patients, such as reluctance to prescribe opioids and insufficient communication with the patient. However, physicians were more likely than nurses to assume that clients over-report their suffering, whereas nursing staff are on the front lines of client care. Their competence in pain assessment allows nurses to provide precise and vital data to responsible physicians for responsible doctors to further pain management [23]. However, physicians are responsible for carefully prescribing medications, including patient evaluation, communications, and teaching [41]. In practice, in the case of opioids, this duty can be amplified. Inappropriate prescription can lead to substantial legal consequences, including malpractice liability, criminal convictions, and medical board discipline [42].

The most frequently observed obstacle related to the patient's lack of awareness of pain management. In contrast, the most frequently observed barrier to the healthcare system was the rigorous restriction of opioid prescription. Because barriers to appropriate pain treatment differ according to whether they have been viewed from the point of view of the client, the treating physician, or the hosting organization; interdisciplinary measures to improve pain management, such as awareness and educational campaigns, must be explored to promote attitudes towards pain treatment [9].

Our study has shown poor practices. Consequently, more than half of the participants have poor practice in controlling critical pain, which corresponds to the study conducted in the northern part of the Amhara region in the northern part of Ethiopia and a study conducted in Uganda [43]. However, participants were more likely to write pain assessments, and this is consistent with previous reports that nursing staff had higher pain assessment skills than doctors and pharmacists [9, 23, 31]. Similarly, most nurses document after assessing the patient’s condition in this study. Research in Europe shows extremely detailed documentation of pain-evaluated and established pain management protocols and procedures [16, 44, 45]. But in Ethiopia, after evaluating pain, around eighty percent of respondents did not document their results [46]. In addition, pain evaluation is provided in Nigeria, although documentation is lacking [47]. Furthermore, the documentation of pain for evaluation, treatment, and reassessment was poor and needs improvement, according to Ayasrah et al. [48].

### Strength and limitation points

To our knowledge, it is the first research of its kind in Palestine, focusing on nurses' knowledge and practices in a wide geographic area, along with reported obstacles to critical illness pain management. However, this study has some limitations; to accurately assess current approaches to managing patient pain, more surveillance and follow-up of nursing practices are required; another possible limitation is the participants' mental and psychological conditions that affect the answer to the questions.

The interpretation of the pain assessment documentation is based on the nurses' perceptions. Therefore, these results may differ from retrospective chart review studies. Finally, the causative relationship between the variables could not have been determined because this was cross-sectional research.

### Relevance to clinical practice

The authors recommend that ICU nurses receive ongoing pain management training based on the study findings. This education should cover pain symptoms, pain assessment, analgesia mechanisms, analgesia evaluation, effects and side effects, and non-pharmacological pain management. The course should be updated regularly.

The authors also recommend that working as a team described the need for collaborative approaches, particularly with opioids, to achieve evidence-based pain management.

In last relevance, intense and comprehensive training in pain management training should become an obligatory component in the nursing curriculum so that student nurses are fully equipped before graduation.

## Conclusions

According to this study, ICU nurses have a knowledge, attitude, and practice gap. Therefore, realistic and updated intervention modules expand nurses’ knowledge and improve attitudes towards effective pain management. In addition, teaching patients and caregivers about pain management is essential since our study found that a lack of knowledge about pain management was the most common obstacle related to patient care.

## Data Availability

The data sets used for the current study are available from the corresponding author upon request.

## Abbreviations

RN: Registered Nurse
LPN: Licensed Practical Nurse
PICU: Pediatric Intensive Care Unit
SICU: Surgical Intensive Care Unit
MICU: Medical Intensive Care Unit
CICU: Cardiac Intensive Care Unit
GICU: General Intensive Care Unit
IQR: Interquartile range
KAPs: Knowledge, attitudes, and practice
SPSS: Statistical Package for the Social Sciences
IRB: Institutional review board
r_s_: Spearman rank correlation
CI: Confidence interval
